# Rituximab plus chemotherapy as first-line treatment in Chinese patients with diffuse large B-cell lymphoma in routine practice: a prospective, multicentre, non-interventional study

**DOI:** 10.1186/s12885-016-2523-7

**Published:** 2016-07-26

**Authors:** Jianqiu Wu, Yongping Song, Liping Su, Li Xu, Tingchao Chen, Zhiyun Zhao, Mingzhi Zhang, Wei Li, Yu Hu, Xiaohong Zhang, Yuhuan Gao, Zuoxing Niu, Ru Feng, Wei Wang, Jiewen Peng, Xiaolin Li, Xuenong Ouyang, Changping Wu, Weijing Zhang, Yun Zeng, Zhen Xiao, Yingmin Liang, Yongzhi Zhuang, Jishi Wang, Zimin Sun, Hai Bai, Tongjian Cui, Jifeng Feng

**Affiliations:** 1 Jiangsu Cancer Hospital, Nanjing, 210000 China; 2 Henan Cancer Hospital, Zhengzhou, China; 3 Shanxi Cancer Hospital, Taiyuan, China; 4 Shanghai Roche Pharmaceuticals Ltd, Shanghai, China; 5 The First Affiliated Hospital of Zhengzhou University, Zhengzhou, China; 6 Jilin University First Affiliated Hospital, Changchun, China; 7 Union Hospital Tongji Medical College Huazhong University of Science and Technology, Wuhan, China; 8 The Second Affiliated Hospital of Zhejiang University School of Medicine, Hangzhou, China; 9 Fourth Hospital of Hebei Medical University, Shijiazhuang, China; 10 Affiliated Hospital of Shandong Academy of Medical Sciences, Jinan, China; 11 Nanfang Medical University Nanfang Hospital, Guangzhou, China; 12 Guangdong Foshan First Hospital, Foshan, China; 13 Guangdong Zhongshan People’s Hospital, Zhongshan, China; 14 Xiangya Hospital Central South University, Changsha, China; 15 Fuzhou General Hospital of Nanjing Military Command, Fuzhou, China; 16 Changzhou First People’s Hospital, Changzhou, China; 17 307 Hospital of PLA, Beijing, China; 18 First Affiliated Hospital of Kunming Medical University, Kunming, China; 19 Affiliated hospital of Neimenggu Medical College, Huhehaote, China; 20 The Fourth Military Medical University Affiliated Tangdu Hospital, Xi’an, China; 21 Daqing General Hospital Group Oilfield General Hospital, Daqing, China; 22 Affiliated Hospital of Guiyang Medical College, Guiyang, China; 23 Anhui Provincial Hospital, Hefei, China; 24 Lanzhou Military Hospital, Lanzhou, China; 25 Fujian provincial hospital, Fuzhou, China

**Keywords:** DLBCL, R-CHOP, Chemotherapy, HBV infection, HBsAg

## Abstract

**Background:**

The efficacy and safety of rituximab-based chemotherapy (R-chemo), the standard regimen for patients with diffuse large B-cell lymphoma (DLBCL), which is more common in Asia than in Western countries, are well confirmed in randomized controlled trials (RCTs). However, the safety and effectiveness of R-chemo in patients who are largely excluded from RCTs have not been well characterized. This real-world study investigated the safety and effectiveness of R-chemo as first-line treatment in Chinese patients with DLBCL.

**Methods:**

Treatment-naive DLBCL patients who were CD20 positive and eligible to receive R-chemo were enrolled with no specific exclusion criteria. Data collected at baseline included age, gender, disease stage, international prognostic index (IPI), B symptoms, extranodal involvement, performance status, and medical history. In the present study, data on safety, treatment effectiveness, and HBV infection management were collected 120 days after the last R-chemo administration.

**Results:**

Overall, R-chemo was well tolerated. The safety profile of R-chemo in patients with a history of heart or liver disease was well described without any additional unexpected safety concerns. The overall response rate (ORR) in the Chinese patients from this study was 94.2 % (complete response [CR], 55.0 %; CR unconfirmed [CRu] 18.2 %; and partial response [PR], 20.9 %). Compared to patients with no history of disease, the CR and PR rates of patients with a history of heart or liver disease were lower and higher, respectively; this tendency could be in part explained by treatment interruptions in patients with heart or liver diseases. HBsAg positivity and a maximum tumor diameter of ≥7.5 cm negatively correlated with CR + CRu, whereas age and HBsAg positivity negatively correlated with CR.

**Conclusions:**

This study further validated the safety and effectiveness of R-chemo in Chinese patients with DLBCL. Patients with a history of heart or liver disease may further benefit from R-chemo if preventive measures are taken to reduce hepatic and cardiovascular toxicity. In addition to IPI and tumor diameter, HBsAg positivity could also be a poor prognostic factor for CR in Chinese patients with DLBCL.

**Trial registration:**

ClinicalTrials.gov #NCT01340443, April 20, 2011.

**Electronic supplementary material:**

The online version of this article (doi:10.1186/s12885-016-2523-7) contains supplementary material, which is available to authorized users.

## Background

Diffuse large B-cell lymphoma (DLBCL) is the most common form of aggressive non-Hodgkin lymphoma (NHL), accounting for approximately 31 % of NHL cases in Western patients [1]. In China, DLBCL is the most common subtype of NHLs (38 %) and mature B-cell neoplasm (54 %) [2]. Currently, rituximab plus chemotherapy (R-chemo) remains the standard of care for patients with DLBCL [3, 4]. The addition of rituximab to chemotherapy significantly improves outcomes in patients with DLBCL, with a 10-year overall survival rate of 43.5 % [5]. Numerous randomized clinical trials (RCTs) have established the benefits of R-chemo in patients with DLBCL [5–10].

However, RCTs have limited generalizability because extrapolation of results is limited to specific groups of patients, as enrolled in the study following stringent eligibility criteria. Patients who are largely excluded from RCTs are more representative of the general population and provide insights into baseline prognostic factors, dosing strategies, management of adverse events (AEs), and treatment effectiveness in real-world settings. Early cardiotoxicity of doxorubicin remains a severe medical concern in DLBCL patients receiving the cyclophosphamide, doxorubicin, vincristine, and prednisolone (CHOP) regimen [11]. Moreover, cardiovascular mortality in patients with lymphoma who receive rituximab with CHOP (R-CHOP) was relatively high, with approximately 30 % of deaths attributed to cardiovascular complications [12]. In addition to cardiotoxicity, the FDA recently issued a warning that patients receiving immune-suppressing rituximab or ofatumumab are at an increased risk of hepatitis B virus (HBV) reactivation [13]. Because HBV infection is highly endemic in China, 12–27 % of all Chinese patients with NHL are positive for hepatitis B surface antigen (HBsAg), which increases the risk of HBV reactivation [14–17]. HBV reactivation may increase hepatic mortality and lead to interruption of curative chemotherapy, which has a deleterious impact on survival outcomes. Despite its clinical importance and urgency, awareness, attitudes, and current screening practices and preventive measures for HBV reactivation among physicians remain suboptimal [18, 19]. A recent study in China reported HBV reactivation in approximately 17.1 % of HBsAg-positive (pos) patients receiving R-chemo [20].

This prospective, non-interventional study evaluated the safety and effectiveness outcomes of R-chemo in real-world clinical settings by including Chinese patients aged ≤18 and >80 years and with history of cardiovascular and liver disease, who were largely excluded from such RCTs. We also investigated HBV infection management in patients with DLBCL.

## Methods

### Study design

This multicenter, single-arm, prospective, non-interventional study is being conducted at 24 centers in China between January 17, 2011 and October 31, 2016. Previously untreated CD20-positive DLBCL patients who were eligible to receive R-chemo (CHOP or non-CHOP) as first-line treatment were enrolled with no specific exclusion criteria. The dose and duration of treatment for each patient was determined at the investigator’s discretion, in accordance with local labeling information (rituximab given at the dose of 375 mg/m^2^ body surface area, once in three weeks) and standard clinical practice. The study was conducted according to the Chinese guidelines for treatment of DLBCL, which was followed by all study centers. Data for baseline characteristics were retrieved from medical records. For the present study, data on safety, treatment effectiveness, and HBV infection management were collected from medical records 120 days after the last rituximab dose administration.

The study protocols were approved by Institutional Review Boards at each center. This study was performed in accordance with Good Clinical Practice and ethical principles of the Declaration of Helsinki. All patients provided written informed consent. The study is registered at clinicaltrials.gov (NCT01340443). Additional information on study procedures has been provided in the Additional file 1.

### Safety and effectiveness assessments

The safety endpoints included AEs, severe adverse events (SAEs), adverse drug reactions (ADRs), and adverse events of special interest (AESIs). The effectiveness endpoints included overall response rate (ORR), complete response (CR), unconfirmed CR (CRu), partial response (PR), progression-free survival (PFS), and overall survival (OS). ORR was defined as the proportion of patients achieving CR, CRu or PR. Treatment response was evaluated using standardized response criteria for NHL [21]. Measurements for assessment were recorded every 2 cycles. Computed tomography (CT) was used to evaluate the lesions and was performed at the investigator’s discretion. The management of HBV was evaluated, including diagnostic techniques for HBV infection and liver function screening prior to R-chemo, monitoring viral replication during and after R-chemo, use of antiviral prophylaxis, and HBV reactivations. Laboratory examinations were performed at the investigator’s discretion in accordance with local clinical practice guidelines. This study reported the safety (AE, SAE, AESI, and ADR) and short-term effectiveness of R-chemo (ORR, CR, CRu, and PR) as well as the management of HBV infection within 120 days after the last R dose administration.

### Statistical analysis

All DLBCL patients who received ≥1 dose of R-chemo were included in the safety analysis populations. Patients who received ≥1 dose of R-chemo and had undergone ≥1 tumor assessment after baseline were evaluable for effectiveness and were included in the intention-to-treat (ITT) population. Descriptive statistics were used to summarize baseline characteristics, HBV infection and replication, and use of antiviral prophylaxis. Demographic data were summarized as mean ± standard deviation for continuous variables and as percentages for categorical variables. Response rates were assessed by calculating percentages and 95 % confidence intervals (CIs) in the ITT population. Categorical variables among subgroups were compared using Fisher’s exact test. Logarithmic transformation was performed for skewed data. Multivariate logistic regression was used to explore association between baseline factors (International Prognostic Index [IPI], age, gender, Eastern Cooperative Oncology Group [ECOG] score, HBsAg/HBcAb, maximum tumor diameter, and history of heart diseases) and treatment responses (CR + CRu and CR), and *p* values <0.05 were considered statistically significant. IPI is an ordered categorical variable categorized as 1 for low risk, 2 for low-intermediate, 3 intermediate-high, and 4 for high. Statistical analyses were conducted using SAS version 9.2.

## Results

### Patient characteristics and treatment

Overall, the safety analysis population included 279 patients with DLBCL. Of these, 258 patients were included in the ITT population, the main reason for exclusion being lack of tumor assessment at baseline. Baseline patient characteristics are summarized in Additional file 1: Table S1.

Baseline characteristics of patients with history of heart disease or liver diseases and patients without disease history are shown in Additional file 1: Table S2. Patients with a history of heart disease were significantly older compared to those without disease history (median age, 68 vs 56 years; *p* < 0.001).

### Safety

In real-world clinical settings, R-chemo was generally well tolerated as first-line treatment in Chinese patients with DLBCL. The incidence of AEs was 95.7 % and the incidence of grade 3–4 AEs, SAEs, AESIs, and ADRs was 52.7, 16.8, 16.5, and 81.0 %, respectively (Table **1**). The most common AEs were low white blood cell count, low neutrophil count, and nausea (Additional file 1: Table S3). Most AEs were resolved through symptomatic treatment, dose reduction, or discontinuation of treatment. The incidence of AE-related deaths was 1.1 % (*n* = 3). Of the 279 patients, 8 (2.9 %) patients discontinued treatment, and 13 (4.7 %) reduced treatment dose due to AEs (Table 2). Table **1** summarizes the age-stratified incidence of AEs, SAEs, AESIs, and ADRs. The most common AEs (any grade and grade 3–4) in patients aged ≤18 or >80 years (*n* = 10) were low white blood cell count and low neutrophil count (Data not shown).

**Table 1:** Summary of AEs, SAEs, AESIs, and ADRs reported in patients receiving R-chemo

Baseline characteristics		AE (any grade), *n* (%)	AE (grade 3–4), *n* (%)	SAE, *n* (%)	AESI, *n* (%)	ADR, *n* (%)
*Total (n = 279)*		267(95.7)	147(52.7)	47(16.8)	46 (16.5)	226 (81.0)
*Age, y*	*19–60 (n = 160)*	206 (95.8)	103 (47.9)	30 (14.0)	32 (14.9)	172 (80.0)
	*61–80 (n = 109)*	22 (95.7)	16 (69.6)	7(30.4)	6 (26.1)	21(91.3)
	*≤18 to >80 (n = 10)*	42 (95.5)	30 (68.2)	11(25.0)	9 (20.5)	36 (81.8)
*History of diseases*	*Heart diseases (n = 23)*	10 (100.0)	5 (50.0)	3 (30.0)	2 (20.0)	10 (100.0)
	*Liver diseases (n = 44)*	152 (95.0)	76 (47.5)	22 (13.8)	22 (13.8)	126 (78.8)
	*No heart or liver diseases (n = 215)*	105 (96.3)	66 (60.6)	22 (20.2)	22 (20.2)	90 (82.6)

*ADR* adverse drug reaction*, AE* adverse event*, AESI* adverse event of special interest*, chemo* chemotherapy*, SAE* severe adverse event*, R* rituximab*, y* year

**Table 2:** Dose reduction and treatment interruptions due to AEs

Treatment interruption	Total (*n* = 279), *n* (%)	No history of heart or liver diseases (*n* = 215), *n* (%)	History of heart diseases (*n* = 23), *n* (%)	History of liver diseases (*n* = 44), *n* (%)	*p* value*	*p* value**
*Dose reduction*	13 (4.7)	9 (4.2)	1 (4.3)	3 (6.8)	1.000	0.435
*Treatment discontinuation*	8 (2.9)	2 (0.9)	2 (8.7)	5 (11.4)	0.048	0.002

*Patients without history of heart or liver diseases were compared with those with history of heart diseases using Fisher’s exact test, and *p* values were obtained

**Patients without history of heart or liver diseases were compared with those with history of liver diseases using Fisher’s exact test, and *p* values were obtained

As mentioned above, 67 patients enrolled in this study had a history of heart or liver disease, characteristics that would normally result in exclusion from RCTs. The incidence of AEs in patients with history of heart or liver diseases was similar to those without disease history (Table **1**). However, patients with a history of heart or liver disease showed an increasing incidence of grade 3–4 AEs, SAEs, and AESIs compared to those without (Table **1**). The most common AEs (System Organ Class-Preferred Terms [SOC-PT]) in patients with history of heart diseases were low white blood cell count, anemia, and nausea (Additional file 1: Table S3). The most common AEs (SOC-PT) in patients with history of liver diseases were low white blood cell count, low neutrophil count, and nausea (Additional file 1: Table S3).

Standardized MedDRA Queries (SMQs) were further applied to identify hepatic and cardiovascular AEs occurring in these patients. A summary of hepatic and cardiovascular AEs (SMQs) occurring in 5 % of the safety analysis population and patients with a history of liver or heart disease is presented in Additional file 1: Table S4 and Additional file 1: Table S5. The incidence of hepatic AEs (SMQ) was 27.3 % (12/44) in patients with history of liver diseases and 22.6 % (63/279) in the safety analysis population (Additional file 1: Table S4). In addition, the incidence rate of cardiovascular AEs (SMQ) was 21.7 % (5/23) in patients with history of heart diseases and 10.4 % (29/279) in the safety analysis population (Additional file 1: Table S5). Grade 3–4 hepatic and cardiovascular AEs were reported in 3.6 % (10/279) and 1.4 % (4/279) of the safety analysis population, respectively. In patients with disease history, grade 3–4 hepatic AEs were reported in 5 of 44 patients with a history of liver disease (11.4 %), whereas grade 3–4 cardiovascular AEs were reported only in 1 of 23 patients with a history of heart disease (4.3 %). The safety profile was similar to that of the safety analysis population, with no addition of unexpected safety concerns. One cardiovascular-related death was reported in the study, which was not related to the study treatment.

In order to determine whether AEs resulted in interruptions of R-chemo treatment in patients with disease history, investigated the incidence of dose reduction and treatment termination due to AEs (Table 2). Two of 23 patients with heart diseases and 5 of 44 patients with liver diseases discontinued therapy due to AEs; one patient with heart disease and 3 with liver diseases had dose reductions due to AEs. The average dose of doxorubicin was numerically lower in patients with heart diseases than those without (79.3 ± 27.11 mg vs 87.5 ± 28.86 mg; *p* = 0.303). The average treatment cycle was similar among patients with a history of heart or liver disease and those without disease history (5.9, 6.1, and 5.9, respectively).

### R-chemo-treated patients with HBV infection

Different HBV infection statuses were defined according to positivity of HBV serological markers (HBsAg and hepatitis B core antibody [HBcAb]): HBsAg-pos group represents patients with active HBV infections or inactive carriers and HBsAg-negative (neg)/HBcAb-pos indicates patients with resolved HBV infections. In this study, 242 (86.7 %) patients were tested for HBsAg and HBcAb prior to DLBCL treatment; of these patients, 9.9 % (24/242) patients were HBsAg-pos, 28.5 % (69/242) were HBsAg-neg/HBcAb-pos, and 61.6 % (149/242) were HBsAg/HBcAb double-neg. For the other 37 patients, HBV infection status at baseline was unknown. Moreover, HBV DNA levels were evaluated at baseline in 70.8 % (17/24) of HBsAg-pos, 44.9 % (31/69) of HBsAg-neg/HBcAb-pos, 18.8 % (28/149) of HBsAg/HBcAb double-neg, and 16.2 % (6/37) of unknown patients. Of these, 47.1 % (8/17) of HBsAg-pos and 3.2 % (1/31) of HBsAg-neg/HBcAb-pos patients were positive for HBV DNA (Additional file 1: Figure S1). At baseline, most patients underwent liver function tests, including alanine aminotransferase (ALT), aspartate aminotransferase (AST), and total bilirubin (TBIL) (Additional file 1: Table S6).

Table 3 presents data on use of antiviral prophylaxis and monitoring of HBV infection. In total, 25/279 patients received antiviral prophylaxis. The proportions of HBsAg-pos and HBsAg-neg/HBcAb-pos patients receiving antiviral prophylaxis were 70.8 % (17/24) and 10.1 % (7/69), respectively. One patient with unknown HBV infection status also received antiviral treatment. Most patients received antiviral treatment at the same time as, or prior to, R-chemo. However, some patients had already discontinued antiviral treatment by 120 days after the last R-chemo dose administration. During R-chemo treatment, HBV serological markers, HBV DNA, and ALT levels were monitored at least once in 43.0, 25.8, and 94.3 % of patients, respectively (Additional file 1: Figure S2). After the last R-chemo dose administration, HBV serological markers, HBV DNA, and ALT levels were monitored at least once in 11.1, 9.3, and 64.2 % of patients, respectively (Additional file 1: Figure S2).

**Table 3:** Use of antiviral prophylaxis and HBV infection management in DLBCL patients receiving R-chemo

Management of HBV infection	HBsAg-pos (*n* = 24)	HBsAg-neg/HBcAb-pos (*n* = 69)	HBsAg/HBcAb double-neg (*n* = 149)	Unknown (*n* = 37)
*HBsAb positivity, n (%)*	3 (12.5)	53 (76.8)	58 (38.9)	1 (2.7)
Antiviral prophylaxis				
*Received antiviral prophylaxis, n (%)*	17 (70.8)	7 (10.1)	0 (0.0)	1 (2.7)
*Median number of cycles administered when prophylaxis was initiated (range)*				
*Time started with prophylaxis relative to R-Chemo (range)*	1 (1, 1)	1 (1, 1)	–	1 (1, 1)
*Stopped prophylaxis by 120 d after last R dose, n (%)*				
*Antiviral treatment duration, day (range)*	−1 (–20, 2)^1^	0 (−1, 0)	–	−4 (−4, −4)
	4 (16.7)	3 (4.3)	–	0
	9 (4–12)^1^	6 (1–185)	–	–
Monitored for serologic markers,^2^ n (%)	14 (58.3)	36 (52.2)	68 (45.6)	2 (5.4)
*Median interval, day (range)*	53.7 (25–205)	37.0 (18–291)	35.3 (21–315)	24.0 (21–27)
*First check after R-chemo, day (range)*	26.0 (1–34)	23.0 (1–61)	19.0 (1–90)	20.0 (20–20)
Monitored for HBV DNA,^3^ n (%)	18 (75.0)	21 (30.4)	15 (10.1)	7 (18.9)
*Median interval, d (range)*	34.9 (22–267)	35.6 (21–168)	40.8 (21–136)	40.0 (22–143)
*First check after R-chemo, d (range)*	21.5 (1–49)	24.0 (1–61)	4.0 (1–25)	21.0 (20–122)
Monitored for liver function,^4^ n (%)	23 (95.8)	65 (94.2)	144 (96.6)	37 (100)
*Median interval, d (range)*	29.9 (20–48)	29.2 (21-51)	28.4 (4–82)	29.0 (15–61)
*First check after R-chemo, d (range)*	26.5 (20–9)	34.0 (8–114)	25.0 (1–117)	22.0 (1–120)

^1^Information on use of antiviral prophylaxis was missing for one subject and was thus not included in the analysis

^2^HBsAg and HBeAg levels were monitored at least twice in the study, including at baseline

^3^HBV DNA was monitored at least twice, including at baseline

^4^ALT levels were monitored at least twice, including at baseline

*chemo,* chemotherapy*; DLBCL,* diffuse large B-cell lymphoma*; HBcAb,* hepatitis B core antibody; *HBsAb,* hepatitis B surface antibody*; HBsAg,* hepatitis B surface antigen*; HBV,* hepatitis B virus; *neg,* negative*; pos,* positive*; R,* rituximab

By the Consensus definition [22, 23], the incidence of HBV reactivation in HBsAg-pos and HBsAg-neg/HBcAb-pos patients was 12.5 % (3/24) and 4.3 % (3/69), respectively. In contrast, investigators only reported 3 cases of HBV reactivation based on their clinical experience: one case each in HBsAg-neg/HBcAb-pos, double-neg, and unknown patients.

### Effectiveness

**Fig. 1:**
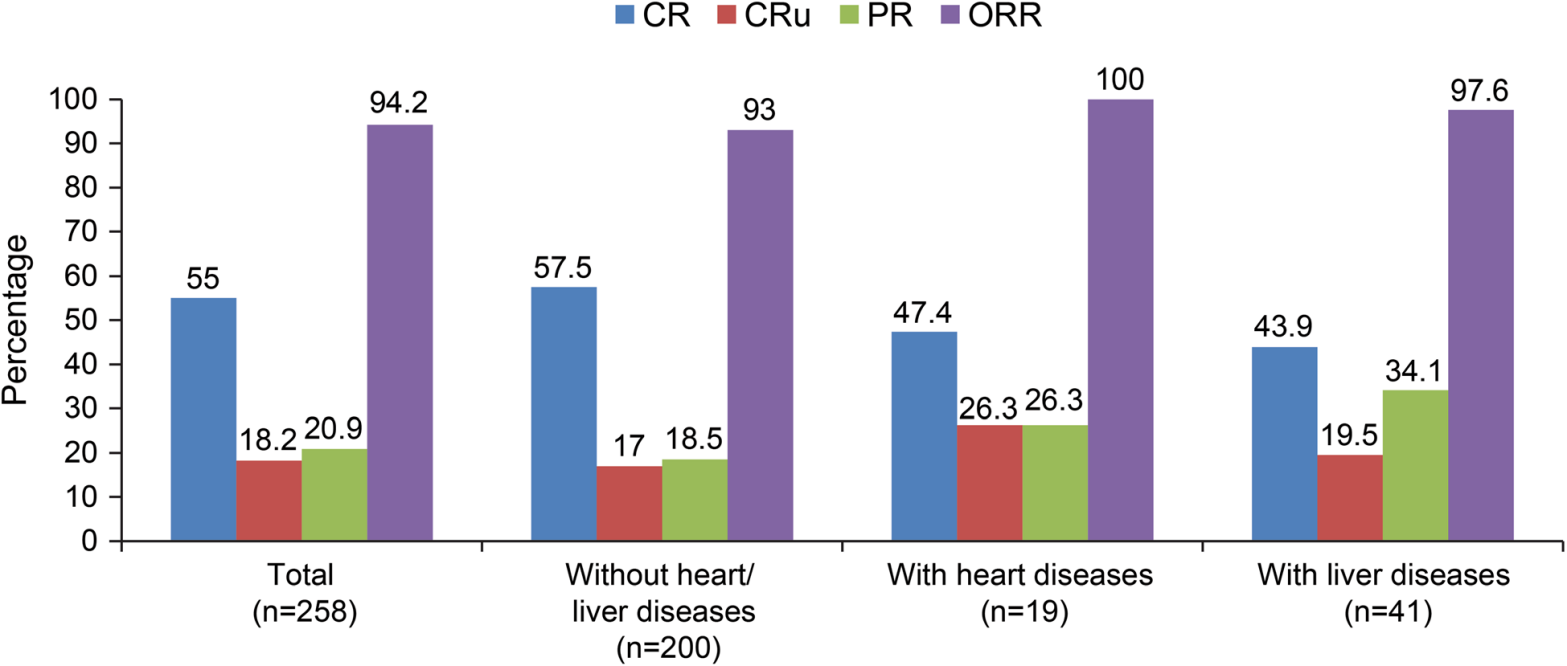
Treatment effectiveness of R-chemo in patients with a history of heart or liver disease (Patients who received ≥1 dose of R-chemo and underwent ≥1 tumor assessments after baseline were included in the analysis *chemo,* chemotherapy; *CR,* complete response*; CRu,* complete response unconfirmed*; PR,* partial response*; ORR,* overall response rate; *R,* rituximab)

In this real-world clinical study, first-line R-chemo treatment in Chinese patients with DLBCL resulted in an ORR of 94.2 % (CR, 55.0 %; CRu, 18.2 %, and PR, 20.9 %). Next, we investigated treatment effectiveness in patients with a history of heart or liver disease. Interestingly, a higher proportion of patients with a history of heart or liver disease achieved PR compared with those without disease history (Fig. 1). In fact, the rate of PR in patients with liver diseases was significantly higher than in those without disease history (34.1 % vs 18.5 %, *p* = 0.035). In contrast, CR rates were lower in patients with a history of heart or liver disease than in those without disease history (47.4 % vs 57.5 %, *p* = 0.470; 43.9 % vs 57.5, *p* = 0.123) (Fig. 1).

**Fig. 2:**
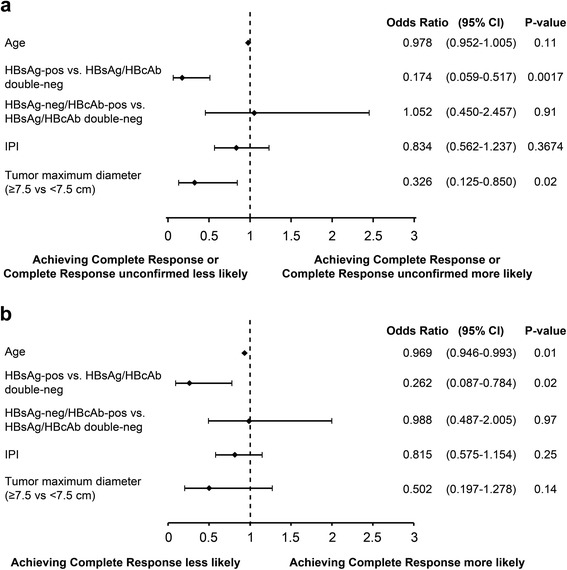
Multivariate logistic regression analyses of prognostic factors. Baseline factors were examined using multivariate logistic regression analyses to investigate prognostic factors of treatment responses. The baseline factors included IPI, age, HBsAg positivity, HBsAg negativity/HBcAb positivity, and maximum tumor diameter. IPI is an ordered categorical variable categorized as 1 for low risk, 2 for low-intermediate, 3 intermediate-high, and 4 for high. **a** Baseline prognostic factors correlated with CR + CRu. **b** Baseline prognostic factors correlated with the likelihood of achieving CR. *CR,* complete response*; CRu,* complete response unconfirmed

To investigate the prognostic factors of treatment, baseline factors were examined. IPI, age, gender, ECOG score, HBsAg/HBcAb, maximum tumor diameter, and history of heart diseases were tested.. HBsAg positivity (*p* = 0.0017; OR < 1) and tumor diameter of ≥7.5 cm (*p* = 0.02; OR < 1) had a negative correlation with CR + CRu (Fig. 2a). In addition, age (*p* = 0.01; OR < 1) or HBsAg positivity (*p* = 0.02; OR < 1) was associated with a reduced likelihood of achieving CR (Fig. 2b). Other baseline factors, such as age, gender, ECOG, HBsAg negativity/HBcAb positivity, or history of heart diseases, were not predictive of CR + CRu or CR.

**Fig. 3:**
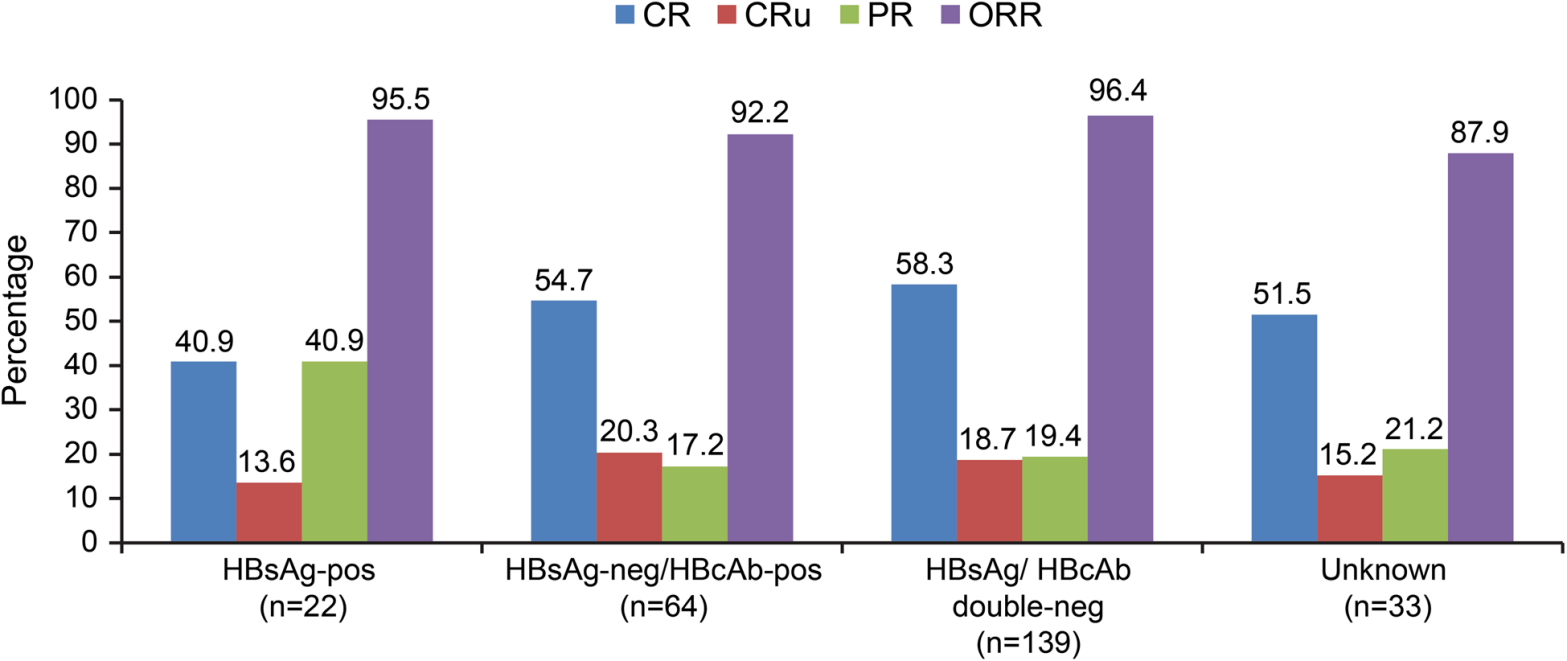
Treatment effectiveness of R-chemo in patients with HBV infectious status (Patients who received ≥1 dose of R-chemo and underwent ≥1 tumor assessment after baseline were included in the analysis *chemo,* chemotherapy*; CR,* complete response*; CRu,* complete response unconfirmed*; PR,* partial response*; ORR,* overall response rate; *R,* rituximab)

Consistent with the multivariate analyses results, the rates of CR and CRu were numerically lower in HBsAg-pos patients than in HBsAg/HBcAb double-neg patients (40.9 % vs 58.3 %, *p* = 0.166; 13.6 % vs 18.7 %, *p* = 0.768) (Fig. 3). In contrast, PR rates were higher in HBsAg-pos patients compared with double-neg patients (40.9 % vs 19.4 %, *p* = 0.050). Further comparisons of baseline factors showed a significant difference in ECOG performance scores between HBsAg-pos patients and double-neg patients (*p* = 0.04; Additional file 1: Table S7). The proportion of HBsAg-pos patients presenting with ECOG scores of 0, 1, 2, and 3 were 9.1, 72.7, 13.6, and 4.5 %, respectively. The median number of treatment cycles was significantly higher in HBsAg-pos patients than in HBsAg/HBcAb double-neg patients (7.5 vs.6.0; *p* = 0.045).

## Discussion

In this real-world clinical study, R-chemo was generally well tolerated in Chinese patients with DLBCL, which further validates the tolerability of R-chemo seen in landmark rituximab trials. The safety profile was well described without unexpected toxicities. Most AEs were resolved through symptomatic treatment, dose reduction, or R-chemo treatment discontinuation. The proportion of AE-related death was 1.1 %. First-line treatment with R-chemo in Chinese patients with DLBCL resulted in an ORR of 94.2 % (CR, 55.0 %; CRu, 18.2 %; and PR, 20.9 %). This is in line with ORRs reported in other studies conducted in Chinese patients [10, 24]. The CR rates were similar between Chinese and Western patients [6]. In a study including Westerners, 8 cycles of R-CHOP treatment produced an ORR of 82.7 % (CR, 52.5 %; CRu, 22.8 %, and PR, 7.4 %) [6], though all patients were aged 60-80 years, 46.0 % had low or low-intermediate risk based on IPI scores and 38.6 % patients had B symptoms. In the present study, Chinese patients with DLBCL tended to be younger (59.7 % patients with age ≤60 years), have low or low-intermediate risk per IPI scores (76.4 %), and fewer patients presented with B symptoms (19.0 %).

In this study, 67 patients had a history of heart or liver disease (23 and 44, respectively), which were the most commonly observed diseases in the patient medical histories. R-chemo was generally well tolerated in these patients; only two patients with heart diseases and five patients with liver diseases discontinued treatment due to AEs. Despite the fact that the incidence of hepatic and cardiovascular AEs was higher in these patients, the safety profile was similar to that of the safety analysis population, with no unexpected toxicities. Interestingly, these patients achieved a higher ORR than those without a history of heart or liver disease. However, these patients had a tendency to achieve PR instead of CR. CR rates were numerically lower in patients with a history of heart or liver disease than in those without. This tendency may be partially attributed to the intrinsic baseline characteristics of these patients and treatment interruptions.

Patients with a history of heart disease (*n* = 23) were older than those without a history of heart or liver disease (68 vs 56 years; *p* < 0.001). The average dose of doxorubicin was numerically lower in patients with heart diseases than in those without (79.3 vs 87.5 mg). Patients with history of heart diseases could further benefit from R-chemo if preventative measures are taken to reduce cardiovascular toxicity.

A total of 44 patients had history of liver diseases, with a significant proportion of patients positive for HBsAg (*n* = 24). In comparison with HBsAg/HBcAb double-neg patients, HBsAg-pos patients achieved lower rates of CR and CRu but significantly higher rates of PR. Moreover, multivariate analyses demonstrate that HBsAg positivity was associated with a reduced likelihood of achieving CR. The tendency of achieving significantly higher PR in HBsAg-pos patients versus double-neg patients was unlikely to be due to inadequate treatment because HBsAg-pos patients received a higher number of R-chemo cycles compared to HBsAg/HBcAb double-neg patients. On the other hand, patients with other HBV infection status (HBsAg-neg/HBcAb-pos) showed similar treatment responses as HBsAg/HBcAb double-neg patients. The mechanism of the negative association between HBsAg and outcomes could be explained in part by the differences in disease presentation. Previous studies have shown that HBsAg-pos patients usually presented with earlier onset and more advanced stages of DLBCL [25]. Indeed, patients reported here showed significantly different presentation of ECOG scores between HBsAg-pos and other groups. Approximately 90.9 % of HBsAg-pos patients presented with ECOG scores ranging 1–3 in contrast to 66.9 % in HBsAg/HBcAb double-neg patients. Consistent with our findings, a recent retrospective study also identified HBsAg positivity as an important risk factor for overall survival of patients with DLBCL receiving R-chemo treatment [26]; in this study, patients with different HBV infection statuses achieved similar outcomes in the CHOP group. However, HBsAg-pos patients in the R-CHOP group presented with unfavorable long-term outcomes compared with uninfected patients and HBsAb-neg/HBcAb-pos patients [26]. Nevertheless, the same study also demonstrated the advantages of R-CHOP over CHOP in treating HBsAg-pos patients; the CR rates were 80.0 % (16/20) for HBsAg-pos patients receiving R-CHOP and 69.4 % (25/36) for those receiving CHOP, indicating benefit of R-CHOP over CHOP in HBsAg-pos patients. However, these findings should be interpreted with caution considering the nature of observational studies and the small sample size. Patients with a history of liver disease, particularly those positive for HBsAg, may further benefit from R-chemo treatment if HBV infection is appropriately managed to reduce hepatic toxicity. Additional studies are warranted to verify the safety and effectiveness of R-chemo in such patients and seek optimal management and treatment regimens. In addition, the present study examined the management of HBV infection in Chinese DLBCL patients in real-world settings. Although it has been established that HBV reactivation in patients receiving chemotherapy can be effectively prevented by the use of antiviral prophylaxis, there remain several unmet needs that hinder optimal management of HBV infection in DLBCL patients. Our study found that most Chinese physicians acknowledged the importance of HBV screening before the initiation of R-chemo. However, improvements in HBV infection monitoring and antiviral treatment are required. Even with monitoring, physicians do not consistently define HBV reactivation, which may lead to underreporting of reactivation, as observed in the present study. Therefore, long-term monitoring is warranted for such patients, particularly after discontinuing antiviral treatment to delay HBV reactivation.

As with observational studies, one of the most significant limitations of the present study was limited availability of data from real-life clinical settings. Observational studies are useful to validate findings from RCTs and draw inferences regarding the safety and effectiveness of treatment but not scientifically capable of proving or disproving hypotheses [27–29]. In addition, the number of very young/old patients and patients with heart or liver diseases in this study were relatively small. Additional studies are warranted to verify these findings. Last, the current study only analyzed safety and effectiveness 120 days after the last dose of rituximab. No study has reported the long-term safety and effectiveness of R-chemo in Chinese patients with DLBCL.

## Conclusions

The results from this study show that the effectiveness and tolerability of R-chemo in real-life clinical practice are in line with those reported in large RCTs. Of note, this study suggests that patients with heart or liver diseases could further benefit from R-chemo if preventative measures are taken to reduce hepatic and cardiovascular toxicity. In addition, this study provides some insights into treatment responses and prognostic factors in Chinese patients with DLBCL. Moreover, HBsAg positivity appeared to be a negative prognostic factor in such patients. Additional studies are warranted to optimize treatment and management strategies in such patients.

## Endnotes

No endnotes were mentioned in the text.

## Abbreviations

ADR, adverse drug reactions; AE, adverse events; AESI, adverse events of special interest; ALT, aminotransferase; AST, aspartate aminotransferase; CHOP, cyclophosphamide, doxorubicin, vincristine and prednisolone; CI, confidence intervals; CR, complete responses; CRu, unconfirmed complete responses; DLBCL, Diffuse large B-cell lymphoma; ECOG, Eastern Cooperative Oncology Group; HBcAb, hepatitis core antibody; HBsAg, hepatitis B surface antigen; HBV, hepatitis B virus; IPI, international prognostic index; ITT, intention-to-treat; Neg, negative; NHL, non-Hodgkin lymphoma; ORR, overall response rates; OS, overall survival; PFS, progression-free survival; Pos, positive; PR, partial responses; R-chemo, rituximab-based chemotherapy; RCT, randomized clinical trials; SAE, severe adverse events; SMQ, Standardized MedDRA Queries; TBIL, total bilirubin

## Additional file

Additional file 1: Table S1. Baseline characteristics. **Table S2**. Comparisons of baseline characteristics between patients with history of heart or liver diseases and patients without heart or liver diseases. **Table S3**. Summary of AEs. **Table S4**. Summary of hepatic AEs. **Table S5**. Summary of cardiovascular AEs. **Table S6**. Summary of screening results of DLBCL patients prior to DLBCL treatment. **Table S7** Comparisons of baseline characteristics between HBsAg-pos or HBsAg-neg/HBcAb-pos patients with double-neg patients. **Figure S1**. HBV DNA testing prior to R-chemo. **Figure S2**. HBV infection monitoring in R-chemo treated DLBCL patients. (DOCX 276 kb)
