# Supplementary material for: Rituximab plus chemotherapy as first-line treatment in Chinese patients with diffuse large B-cell lymphoma in routine practice: a prospective, multicentre, non-interventional study
Source: BMC Cancer. 2016 Jul 26;16:537. doi: 10.1186/s12885-016-2523-7 (PMC4962436; doi:10.1186/s12885-016-2523-7)
Supplement: Additional file 1: Table S1. — Baseline characteristics. Table S2. Comparisons of baseline characteristics between patients with history of heart or liver diseases and patients without heart or liver diseases. Table S3. Summary of AEs. Table S4. Summary of hepatic AEs. Table S5. Summary of cardiovascular AEs. Table S6. Summary of screening results of DLBCL patients prior to DLBCL treatment. Table S7 Comparisons of baseline characteristics between HBsAg-pos or HBsAg-neg/HBcAb-pos patients with double-neg patients. Figure S1. HBV DNA testing prior to R-chemo. Figure S2. HBV infection monitoring in R-chemo treated DLBCL patients. (DOCX 276 kb) [file 12885_2016_2523_MOESM1_ESM.docx]

# Supplementary Methods

**Study design**

The diagnosis of DLBCL was confirmed by biopsy according to the World Health Organization classification. The dose and duration of treatment for each patient was at the discretion of the investigator, in accordance with local labelling information and clinical practice. Patients were observed throughout the duration of R-chemo treatment and will be followed for up to 3 years post-treatment. Data collected at baseline included age, gender, disease stage, international prognostic index (IPI), B symptoms, extranodal involvement, performance status and past medical history. Performance statuses were assessed according to the Eastern Cooperative Oncology Group performance score (ECOG PS) criteria.

**Safety and effectiveness assessments**

Grading of AEs was performed according to the National Cancer Institute (NCI) Common Terminology Criteria for Adverse Events (CTC-AE), version 4.0 and Standardized MedDRA Queries (SMQ). PFS was defined as the time from receiving the first dose of treatment until disease progression or death. OS was defined as the time from receiving the first dose of treatment until death as a result of any cause.

**HBV infection management**

The management of HBV was evaluated, including HBV infection and liver function screening prior to R-chemo, viral replication monitoring during and after R-chemo, antiviral prophylaxis use and HBV reactivations. Laboratory assessments were performed at the discretion of the investigator, according to local clinical practice and guidelines. Results of hepatitis B panel tests (HBsAg, HBsAb, HBeAg, HBeAb, HBcAb), HBV DNA assays, liver function tests [(aminotransferase [ALT], aspartate aminotransferase [AST], total bilirubin [TBIL]) were collected. Different HBV infection statuses were defined according to positivity of HBsAg and HBcAb: HBsAg-positive (pos) represents patients with active HBV infections or inactive carrier state, HBsAg-negative (neg)/ HBcAb-positive (pos) patients indicate patients with resolved HBV infections. HBV reactivation was defined according to the Chinese consensus on management of lymphoma patients with HBV infection. In brief, HBsAg-pos patients had HBV reactivation if HBV DNA increased ≥ 1 log10 from baseline, if HBV DNA appeared (above the lower limit of detection) or if HBeAg appeared in HBeAg-neg patients. HBsAg-neg/HBcAb-pos patients had HBV reactivation if there was appearance of either HBsAg or HBV DNA (above the lower limit of detection).

# Supplementary Table 1. Baseline characteristics

| Variables | SAS (n=279), n (%) | | ITT (n=258), n (%) | |
| --- | --- | --- | --- | --- |
|  | **N** | **%** | **n** | **%** |
| Male | 162 (58.1) | | 148 (57.4) | |
| Age, years | | | | |
| Median (range), years | 57.2 (12.8–88.4) | | 57.2 (12.8–88.4) | |
| ≤18 | 4 (1.4) | | – | |
| 19–60 | 160 (57.3) | | – | |
| ≤60 | – | | 154 (59.7) | |
| 61–80 | 109 (39.1) | | 98 (38.0) | |
| >80 | 6 (2.2) | | 6 (2.3) | |
| Stage | | | | |
| I | 42 (15.1) | | 38 (14.8) | |
| II | 92 (33.0) | | 86 (33.5) | |
| III | 68 (24.5) | | 66 (25.6) | |
| IV | 76 (27.3) | | 67 (26.1) | |
| Missing data^1^ | 1 | | 1 | |
| ECOG | | | | |
| 0 | 71 (25.4) | | 68 (26.4) | |
| 1 | 179 (64.2) | | 167 (64.7) | |
| 2 | 21 (7.5) | | 17 (6.6) | |
| 3 | 7 (2.5) | | 6 (2.3) | |
| 4 | 1 (0.4) | | 0 (0.0) | |
| IPI | | | | |
| Low risk | 140 (50.5) | | 131 (51.2) | |
| Low-Intermediate risk | 71 (25.6) | | 66 (25.8) | |
| Intermediate-high risk | 46 (16.6) | | 43 (16.8) | |
| High risk | 20 (7.2) | | 16 (6.3) | |
| Missing data^1^ | 2 | | 2 | |
| B symptoms | 52 (18.6) | | 49 (19.0) | |
| Bulky disease |  | | | |
| Tumor maximum  diameter ≥ 7.5cm | 30 (13.5) | | 29 (13.9) | |
| Missing data^*^ | 57 | | 49 | |
| Extranodal sites |  | | | |
| ≥1 | 153 (55.6) | | 140 (54.9) | |
| Missing data^1^ | 4 | | 3 | |

*^1^Patients with missing data were excluded from the analysis. SAS: safety analysis; ITT: intention-to-treat; ECOG: Eastern Cooperative Oncology Group; IPI: international prognostic index.*

# Supplementary Table 2. Comparisons of baseline characteristics between patients with history of heart or liver diseases and patients without heart or liver diseases.

| Baseline characteristics | | Patients with heart diseases  (n=19),  n (%) | Patients with liver diseases (n=41),  n (%) | Patients without heart or liver diseases (n=200),  n (%) | p-value^1^ | p-value^2^ |
| --- | --- | --- | --- | --- | --- | --- |
| Age | ≤ 60 y | 2 (10.5) | 26 (63.4) | 127 (63.5) | <0.001 | 0.833 |
|  | 61– 80 y | 16 (84.2) | 15 (36.6) | 68 (34.0) |  |  |
|  | >80 y | 1 (5.3) | 0 (0.0) | 5 (2.5) |  |  |
| IPI | Low risk | 7 (36.8) | 19 (47.5) | 106 (53.3) | 0.433 | 0.860 |
|  | Low/Intermediate risk | 6 (31.6) | 12 (30.0 | 48 (24.1) |  |  |
|  | Intermediate/high risk | 5 (26.3) | 7 (17.5) | 32 (16.1) |  |  |
|  | High risk | 1 (5.3) | 2 (5.0) | 13 (6.5) |  |  |
|  | Missing data^3^ | 0 | 1 | 1 |  |  |
| Stage | I | 4 (21.1) | 5 (12.2) | 29 (14.6) | 0.573 | 0.600 |
|  | II | 6 (31.6) | 12 (29.3) | 69 (34.7) |  |  |
|  | III | 6 (31.6) | 14 (34.1) | 47 (23.6) |  |  |
|  | IV | 3 (15.8) | 10 (24.4) | 54 (27.1) |  |  |
|  | Missing data^3^ | 0 | 0 | 1 |  |  |
| ECOG | 0 | 2 (10.5) | 12 (29.3) | 54 (27.0) | 0.221 | 0.647 |
|  | 1 | 15 (78.9) | 24 (58.5) | 130 (65.0) |  |  |
|  | 2 | 1 (5.3) | 4 (9.8) | 12 (6.0) |  |  |
|  | 3 | 1 (5.3) | 1 (2.4) | 4 (2.0) |  |  |
| Tumor maximum diameter | <7.5 cm | 13 (86.7) | 31 (91.2) | 138 (85.2) | 1.000 | 0.583 |
|  | ≥7.5 cm | 2 (13.3) | 3 (8.8) | 24 (14.8) |  |  |
|  | Missing data^3^ | 4 | 7 | 38 |  |  |
| Extranodal involvement | ≥1 | 10 (52.6) | 23 (56.1) | 108 (54.8) | 1.000 | 1.000 |
|  | 0 | 9 (47.4) | 18 (43.9) | 89 (45.2) |  |  |
|  | Missing data^3^ | 0 | 0 | 3 |  |  |

*^1^Baseline characteristics were compared between patients with history of heart diseases and patients without heart or liver diseases. ^2^Baseline characteristics were compared between patients with history of liver diseases and patients without heart or liver diseases. ^3^Patients with missing data were excluded from the analysis.* *ECOG: Eastern Cooperative Oncology Group; IPI: international prognostic index.*

# Supplementary Table 3. Summary of AEs

| System Organ Class (SOC) and Preferred Terms (PT) | Total (N=279),  n (%) | Heart diseases (n=23), n (%) | Liver diseases (n=44), n (%) |
| --- | --- | --- | --- |
| AE (any grade) ≥5% | | | |
| Low white blood cell count | 139 (49.8) | 13 (56.5) | 19 (43.2) |
| Low neutrophil count | 61 (21.9) | 3 (13.0) | 13 (29.5) |
| Nausea | 50 (17.9) | 6 (26.1) | 9 (20.5) |
| Anemia | 48 (17.2) | 9 (39.1) | 7 (15.9) |
| Upper respiratory tract infection | 41 (14.7) | 3 (13.0) | – |
| Pulmonary infection | 34 (12.2) | 3 (13.0) | – |
| Infectious pneumonia | 31 (11.1) | 4 (17.4) | – |
| Fever | 29 (10.4) | – | 9 (20.5) |
| Low platelet count | 24 (8.6) | 3 (13.0) | 8 (18.2) |
| Bone marrow failure | 23 (8.2) | – | 4 (9.1) |
| Gastrointestinal dysfunction | 23 (8.2) | – | 5 (11.4) |
| Diahhrea | 22 (7.9) | – | 6 (13.6) |
| Liver dysfunction | 20 (7.2) | – | – |
| Constipation | 17 (6.1) | 5 (21.7) | – |
| Fatigue | 17 (6.1) | – | – |
| Infusion-related reactions | 17 (6.1) | – | 5 (11.4) |
| Low hemoglobin count | 15 (5.4) | – | – |
| Vomiting | 15 (5.4) | – | – |
| Cough | 14 (5.0) | – | – |
| Low granulocyte count | – | – | 6 (13.6) |
| Granulocytopenia | 14 (5.0) | – | 4 (9.1) |
| Neutropenia | – | 2 (8.7) | – |
| Hair loss | – | – | 3 (6.8) |
| Chills | – | – | 4 (9.1) |
| AE (grade 3–4) ≥ 5% | | | |
| Low white blood cell count | 59 (21.1) | 8 (34.8) | 10 (22.7) |
| Upper respiratory tract infection | 41 (14.7) | – | – |
| Low neutrophil count | 32 (11.5) | 2 (8.7) | 7 (15.9) |
| Pulmonary infection | 20 (7.2) | 2 (8.7) | – |
| Low platelet count | 16 (5.7) | – | 5 (11.4) |
| Bone marrow failure | 14 (5.0) | – | 3 (6.8) |
| Neutropenia | – | 2 (8.7) | – |
| SAE ≥ 5% | | | |
| Pulmonary infection | 19 (6.8) | 3 (13.0) | – |

*AE: adverse event; SAE: severe adverse event.*

# Supplementary Table 4. Summary of hepatic AEs

|  | Any grade, n (%) | | Grade 3–4, n (%) | | SAE, n (%) | | Death, n (%) | |
| --- | --- | --- | --- | --- | --- | --- | --- | --- |
|  | **Total (N=279)** | **Liver diseases (n=44)** | **Total (N=279)** | **Liver diseases (n=44)** | **Total (N=279)** | **Liver diseases (n=44)** | **Total (N=279)** | **Liver diseases (n=44)** |
| ≥5% | **63 (22.6)** | **12 (27.3)** | **10 (3.6)** | **5 (11.4)** | **2 (0.7)** | **2 (4.5)** | **0 0.0** | **0 (0.0)** |
| Drug related hepatic disorders | 59 (21.1) | 9 (20.5) | 7 (2.5) | 3 (6.8) | 0 (0.0) | 0 (0.0) | 0 (0.0) | 0 (0.0) |
| Severe events only | 14 (5.0) | 3 (6.8) | 4 (1.4) | 2 (4.5) | 0 (0.0) | 0 (0.0) | 0 (0.0) | 0 (0.0) |
| Hepatic failure: fibrosis and cirrhosis and other liver damage-related conditions | 13 (4.7) | 3 (6.8) | 3 (1.1) | 2 (4.5) | 0 (0.0) | 0 (0.0) | 0 (0.0) | 0 (0.0) |
| Liver related investigations, signs and symptoms | 47 (16.8) | 6 (13.6) | 3 (1.1) | 1 (2.3) | 0 (0.0) | 0 (0.0) | 0 (0.0) | 0 (0.0) |
| Liver infections | 5 (1.8) | 3 (6.8) | 3 (1.1) | 2 (4.5) | 0 (0.0) | 2 (4.5) | 0 (0.0) | 0 (0.0) |

*AE: adverse event; SAE: severe adverse event; SMQ: Standardized MedDRA Queries.*

# Supplementary Table 5. Summary of cardiovascular AEs

| Cardiovascular AEs (SMQ terms) | Any grade, n (%) | | Grade 3–4, n (%) | | | SAE, n (%) | | | Death, n (%) | |
| --- | --- | --- | --- | --- | --- | --- | --- | --- | --- | --- |
|  | **Total (N=279)** | **Heart diseases (n=23)** | | **Total (N=279)** | **Heart diseases (n=23)** | | **Total (N=279)** | **Heart diseases (n=23)** | **Total (N=279)** | **Heart diseases (n=23)** |
| ≥5% | 29 (10.4) | 5 (21.7) | | 4 (1.4) | 1 (4.3) | | 3 (1.1) | 1 (4.3) | 1 (0.4) | 1 (4.3) |
| Cardiac arrhythmias | 24 (8.6) | 3 (13.0) | | 2 (0.7) | 0 (0.0) | | 1 (0.4) | 0 (0.0) | 0 (0.0) | 0 (0.0) |
| Arrhythmia related investigations, signs and symptoms | 11 (3.9) | – | | 0 (0.0) | – | | 0 (0.0) | – | 0 (0.0) | – |
| *Heart palpitations* | 9 (3.2) | – | | 0 (0.0) | – | | 0 (0.0) | – | 0 (0.0) | – |
| Cardiac arrhythmia terms | 13 (4.7) | 2 (8.7) | | 2 (0.7) | 0 (0.0) | | 1 (0.4) | 0 (0.0) | 0 (0.0) | 0 (0.0) |
| *Ventricular extrasystole* | 3 (1.1) | – | | 0 (0.0) | – | | 0 (0.0) | – | 0 (0.0) | – |
| *Arrhythmia* | 4 (1.4) | – | | 1 (0.4) | – | | 1 (0.4) | – | 0 (0.0) | – |
| Cardiac failure | 6 (2.2) | 3 (13.0) | | 2 (0.7) | 1 (4.3) | | 2 (0.7) | 1 (4.3) | 1 (0.4) | 1 (4.3) |
| Peripheral edema | 4 (1.4) | 2 (8.7) | | 0 (0.0) | 0 (0.0) | | 0 (0.0) | 0 (0.0) | 0 (0.0) | 0 (0.0) |
| Cardiac failure | 2 (0.7) | – | | 1 (0.4) | – | | 2 (0.7) | – | 1 (0.4) | – |
| Cardiomyopathy | 19 (6.8) | 3 (13.0) | | 3 (1.1) | 1 (4.3) | | 3 (1.1) | 1 (4.3) | 1 (0.4) | 1 (4.3) |
| Heart palpitation | 9 (3.2) | – | | 0 (0.0) | – | | 0 (0.0) | – | 0 (0.0) | – |
| Cardiac failure | 2 (0.7) | – | | 1 (0.4) | – | | 2 (0.7) | – | 1 (0.4) | – |
| Arrhythmia | 4 (1.4) | – | | 1 (0.4) | – | | 1 (0.4) | – | 0 (0.0) | – |

*AE: adverse event; SAE: severe adverse event; SMQ: Standardized MedDRA Queries.*

# Supplementary Table 6. Summary of screening results of DLBCL patients prior to DLBCL treatment

|  | HBsAg-pos  (N=24), n/N (%) | HBsAg-neg/  HBcAb-pos  (N=69), n/N (%) | HBsAg/HBcAb double-neg (N=149),  n/N (%) | Unknown (N=37), n/N (%) |
| --- | --- | --- | --- | --- |
| ALT normal | 20/24 (83.3) | 60/69 (87.0) | 132/147 (89.8) | 31/36^1^ (86.1) |
| AST normal | 20/24 (83.3) | 60/68^1^ (88.2) | 139/147 (94.6) | 35/36^1^ (97.2) |
| TBIL normal | 22/24 (91.7) | 67/68^1^ (98.5) | 130/147 (88.4) | 33/35^2^ (94.3) |

*^1^One patient has missing data and was excluded from the analysis.* *^2^Two patients have missing data and were excluded from the analysis. DLBCL: diffuse large B-cell lymphoma; ALT: aminotransferase; AST: aspartate aminotransferase; TBIL: total bilirubin; HBsAg: hepatitis B surface antigen; pos: positive; neg: negative; HBcAb: hepatitis B core antibody.*

# Supplementary Table 7 Comparisons of baseline characteristics between HBsAg-pos or HBsAg-neg/HBcAb-pos patients with double-neg patients.

| Baseline characteristics | | HBsAg-pos  (n=22),  n (%) | HBsAg-neg/HBcAb-pos (n=64),  n (%) | double neg (n=139),  n (%) | p-value^1^ | p-value^2^ |
| --- | --- | --- | --- | --- | --- | --- |
| Age | ≤ 60 y | 16 (72.7) | 36 (56.3) | 75 (54.0) | 0.262 | 0.761 |
|  | 61– 80 y | 6 (27.3) | 26 (40.6) | 61 (43.9) |  |  |
|  | >80 y | 0 (0.0) | 2 (3.1) | 3 (2.2) |  |  |
| IPI | Low risk | 12 (54.5) | 34 (53.1) | 66 (47.8) | 0.912 | 0.197 |
|  | Low/Intermediate risk | 5 (22.7) | 11(17.2) | 42 (30.4) |  |  |
|  | Intermediate/high risk | 4 (18.2) | 13 (20.3) | 22 (15.9) |  |  |
|  | High risk | 1 (4.5) | 6 (9.4) | 8 (5.8) |  |  |
|  | Missing data^‡^ | 0 | 0 | 1 |  |  |
| Stage | I | 1 (4.5) | 13 (20.6) | 21 (15.1) | 0.667 | 0.023 |
|  | II | 8 (36.4) | 24 (38.1) | 45 (32.4) |  |  |
|  | III | 7 (31.8) | 6 (9.5) | 39 (28.1) |  |  |
|  | IV | 6 (27.3) | 20 (31.7) | 34 (24.5) |  |  |
|  | Missing data^3^ | 0 | 1 | 0 |  |  |
| ECOG | 0 | 2 (9.1) | 17 (26.6) | 46 (33.1) | 0.040 | 0.640 |
|  | 1 | 16 (72.7) | 39 (60.9) | 82 (59.0) |  |  |
|  | 2 | 3 (13.6) | 6 (9.4) | 8 (5.8) |  |  |
|  | 3 | 1 (4.5) | 2 (3.1) | 3 (2.2) |  |  |
| Tumor maximum diameter | <7.5 cm | 15 (83.3) | 43 (87.8) | 99 (86.1) | 0.722 | 1.000 |
|  | ≥7.5 cm | 3 (16.7) | 6 (12.2) | 16 (13.9) |  |  |
|  | Missing data^3^ | 4 | 15 | 24 |  |  |
| Extranodal involvement | ≥1 | 9 (40.9) | 31 (48.4) | 78 (56.5) | 0.249 | 0.293 |
|  | 0 | 13 (59.1) | 33 (51.6) | 60 (43.5) |  |  |
|  | Missing data^3^ | 0 | 0 | 1 |  |  |

*^1^Baseline characteristics were compared between HBsAg-pos and double neg patients. ^2^Baseline characteristics were compared between HBsAg-pos and HBsAg-neg/HBcAb-pos patients. ^3^Patients with missing data were excluded from the analysis.* *ECOG: Eastern Cooperative Oncology Group; IPI: international prognostic index.*

# Supplementary **Figure 1. HBV DNA testing prior to R-chemo.** Rates of HBV DNA testing prior to R-chemo and positive HBV DNA in HBsAg-pos, HBsAg-neg/HBcAb-pos, HBsAg/HBcAb double-neg and unknown patients are shown in the clustered bar chart. Rates of HBV DNA testing were expressed by the number of patients tested over the total number of patients in each subgroup. Rates of positive HBV DNA were expressed by the number of patients who were HBV DNA positive (above the upper detection limit of the PCR kit) over the total number of tested patients in each subgroup. R: rituximab; chemo: chemotherapy; HBV: hepatitis B virus; HBsAg: hepatitis surface antigen; neg: negative; pos: positive; HBcAb: hepatitis B core antibody.

**
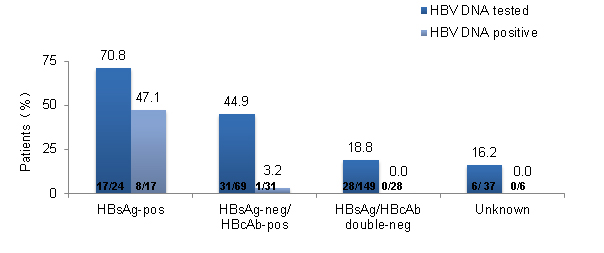
**

# Supplementary Figure 2. **HBV infection monitoring in R-chemo treated DLBCL patients.** The proportion of patients who received monitoring in each subgroup is indicated at the top of each bar and the number of patients indicated at the bottom of each bar. (A) Proportions of patients monitored for serologic markers (HBsAg and HBeAg) during R-chemo and after R-chemo. (B) Proportions of patients monitored for HBV DNA during R-chemo and after R-chemo. (C) Proportions of patients monitored for liver function (ALT) during R-chemo and after R-chemo. R: rituximab; chemo: chemotherapy; DLBCL: diffuse large B-cell lymphoma; HBV: hepatitis B virus; HBsAg: hepatitis surface antigen; neg: negative; pos: positive; HBcAb: hepatitis B core antibody; ALT: aminotransferase.

**
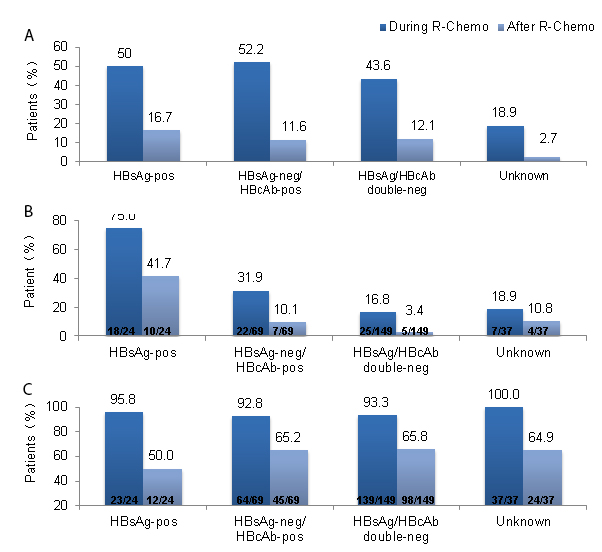
**
